# The Illogicality of Stock-Brokers: Psychological Experiments on the Effects of Prior Knowledge and Belief Biases on Logical Reasoning in Stock Trading

**DOI:** 10.1371/journal.pone.0013483

**Published:** 2010-10-18

**Authors:** Markus Knauff, Claudia Budeck, Ann G. Wolf, Kai Hamburger

**Affiliations:** Experimental Psychology and Cognitive Science, Justus Liebig University Giessen, Giessen, Germany; University of Granada, Spain

## Abstract

**Background:**

Explanations for the current worldwide financial crisis are primarily provided by economists and politicians. However, in the present work we focus on the psychological-cognitive factors that most likely affect the thinking of people on the economic stage and thus might also have had an effect on the progression of the crises. One of these factors might be the effect of prior beliefs on reasoning and decision-making. So far, this question has been explored only to a limited extent.

**Methods:**

We report two experiments on logical reasoning competences of nineteen stock-brokers with long-lasting vocational experiences at the stock market. The premises of reasoning problems concerned stock trading and the experiments varied whether or not their conclusions—a proposition which is reached after considering the premises—agreed with the brokers' prior beliefs. Half of the problems had a conclusion that was highly plausible for stock-brokers while the other half had a highly implausible conclusion.

**Results:**

The data show a strong belief bias. Stock-brokers were strongly biased by their prior knowledge. Lowest performance was found for inferences in which the problems caused a conflict between logical validity and the experts' belief. In these cases, the stock-brokers tended to make logically invalid inferences rather than give up their existing beliefs.

**Conclusions:**

Our findings support the thesis that cognitive factors have an effect on the decision-making on the financial market. In the present study, stock-brokers were guided more by past experience and existing beliefs than by logical thinking and rational decision-making. They had difficulties to disengage themselves from vastly anchored thinking patterns. However, we believe, that it is wrong to accuse the brokers for their “malfunctions”, because such hard-wired cognitive principles are difficult to suppress even if the person is aware of them.

## Introduction

Beginning in 2007 and continuing through the years 2008 to 2010 the finical markets around the world experienced the first crisis of the new millennium. The crunch was caused by a subprime mortgage crisis in the United States and many malfunctions of the financial systems around the globe. The result of the crash was that between January and October 2008, stock owners in the U.S. had suffered about $8 trillion in losses and losses in other countries have averaged about 40% (Wall Street Journal, October 11, 2008, p.1). How could that happen? In the media mainly economists and politicians voice their opinion on the causes of and possible solutions for the crisis and try to explain the breakdown, for instance, as a result of a globalized world or attribute it to greediness and moral irresponsibility of the people in charge. In support of the latter assertion, *The Economist* titled an article on the future of the financial markets “Greed—and Fear” (The Economist, January 24, 2009) and the Frankfurter Allgemeine Zeitung, the leading newspaper in Germany, under the rubric “glossary of the crises” entitled an article on the most popular explanations for the crisis with a single word: Gier (engl. “Greed”; FAZ, May 12, 2009).

While in the public discourse greed and immorality are very popular psychological concepts to explain the financial breakdown, other psychological factors that probably caused or, at least, triggered the crises are almost completely neglected.

In the present work we focus on the cognitive factors that most likely affect the thinking of people on the economic stage and thus might also have had an effect on the progression of the crises. The main assumption of the paper is that fatal decisions and inappropriate actions on the finical market can also be caused by the “natural” and almost “hard-wired” limitations of the human cognitive system. An important factor in this context might be the effect of prior knowledge and existing beliefs. Experts in a certain domain typically possess domain-specific skills and knowledge that distinguish them from novices and less experienced people in the domain. As a consequence they typically solve problems more quickly and more accurately than laymen [1], [2]. They analyse a given problem in different ways than non-experts and use different cognitive strategies to solve problems compared to novices [3], [4]. Typically, this results in better performance of the experts (for an overview on the psychology of expertise see [5], [6]) and often results in their enormous salaries.

However, prior knowledge and existing beliefs can also be a drawback in reasoning and decision-making. When we are an expert in a specific domain and we are convinced that something is true, we often have serious problems in changing our mind in situations where what we think is true is actually wrong. It is difficult to detect the inconsistency of our prior experiences and knowledge with the current situation and it also proves hard to revise our beliefs in order to take a new piece of information into account. Findings from cognitive brain research also provide evidence that reasoning with knowledge-related problems is implemented in other brain areas than reasoning with abstract materials with no meaningful content [7], [8]. Reasoning in general activates a large bilateral network including occipital, parietal, temporal and frontal lobes, basal ganglia, and cerebellar regions. Which of these areas are involved highly depends on the type of the problems we are confronted with [9]–[12]. In particular, further investigations reveal that reasoning about problems related to prior beliefs activates a left frontal (BA 47) temporal (BA 21/22) system, whereas reasoning about problems in unfamiliar domains activates bilateral parietal lobes (BA 7, 40) and dorsal PFC (BA 6).

One of the most impressive findings in this context is the belief bias [13]. A belief bias typically occurs when prior knowledge significantly influences how a reasoning problem is solved. Technically speaking, a logically valid inference is one whose conclusion is true in every case in which all its premises are true [14]. However, the conclusion of such an inference can be true or false in relation to our prior knowledge. If the conclusion is true with respect to our prior knowledge the inference is supported. If it is false in relation to our prior knowledge the inference is more difficult, which means that it results in more errors or longer decision times.

Goel and Dolan [7] also identified the neural basis of the belief bias. They brought logic reasoning and beliefs into conflict and found evidence for the engagement of a left temporal lobe system during belief-based reasoning and a bilateral parietal lobe system during belief-neutral reasoning. Activation of the right prefrontal cortex was found when the participants inhibited a response associated with belief bias and correctly completed a logical task. When logical reasoning, in contrast, was overwritten by a belief bias, there was engagement of the ventral medial prefrontal cortex, a region implicated in affective processing.

The present contribution explored how stock-brokers perform when they are confronted with problems that evoke a clash between their prior beliefs and what would be a logically valid inference. Particularly, we were interested in problems where a conflict occurred between what the brokers have known to be true as a general rule for calculating stock prices and what a logically correct inference would be.

## Methods

### Ethics statement

The experiments reported here were done in accordance with the Declaration of Helsinki and followed the ethical requirements of the German Psychological Association (DGPs). No extra ethical approval was required for this study, since the material was harmless and dealt with work content of stock-brokers. Participants were informed that their data is treated anonymously and that they could terminate the experiment at any time without providing any reason. All participants provided informed written consent.

### Experiment 1

#### Participants

Nineteen experienced stock-brokers (with the majority having over ten years of experience on the trading floor), working in large finance companies on the Frankfurt stock market, were tested. Their age ranged between 24 and 65 years.

#### Materials and procedure

**Figure 1:**
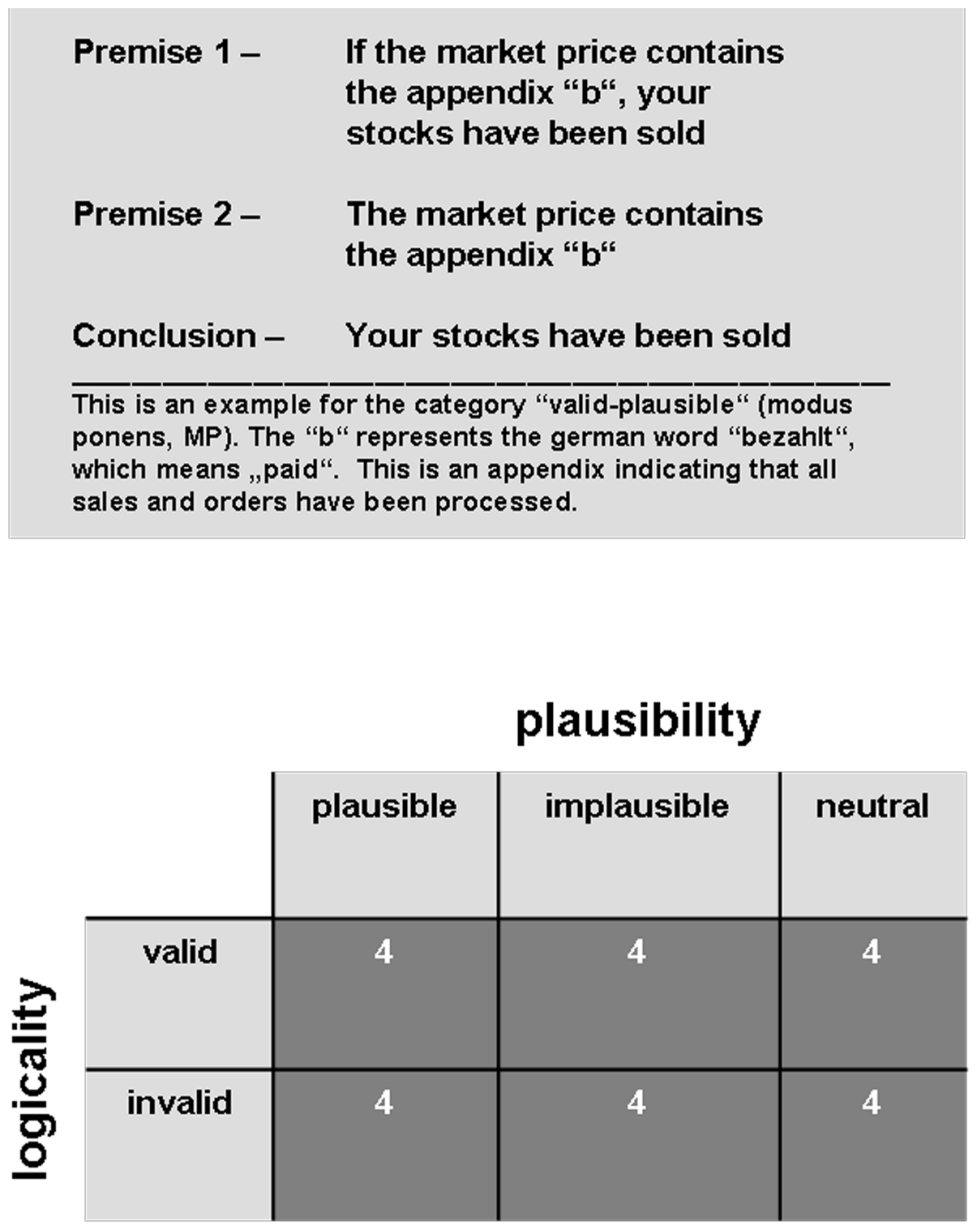
Design of the Experiment. Sample item showing an inference of the MP type (top). Experimental paradigm (bottom). See text for details.

The experiment was conducted on a laptop computer that presented the problems and recorded participants' responses. The stock-brokers judged the validity of 24 logical inference problems concerning sales transactions and calculating stock prices (Figure 1 top). This content was integrated into reasoning problems that consisted of two premises and one conclusion. The premises are the statements that the participants had to take for granted (although they might conflict with their prior knowledge) and the conclusion was the statement that had to be deducted from the premises. All problems were conditional inferences, consisting of an “if A then B” construct that posits B to be true if A is true. The four common inference problems were used: Modus Ponens (MP), Modus Tollens (MT), Denial of the Antecedent (DA), and Affirmation of the Consequent (AC). Logically, only MP (if a then b; a; b) and MT (if a then b; not-b; not-a) are valid inferences, whereas DA (if a then b; not-a; not-b) and AC (if a then b; b; a) are logically invalid [10]. Although we did not predict specific differences between the different types of inferences, we used MP, MT, DA, and AC problems to see whether they are differently affected by the content of the problem and also to design our material in accordance with the standards in reasoning research. The participants were instructed to use the normal interpretation of conditionals, i.e., not to interpret them as biconditional (which would make the DA, and AC inferences logically valid, too). The formal validity of an inference was checked against the laws of formal logic that allows to exactly determine what is logically valid. Participants' decisions were analysed with respect to logical correctness and agreement with the experts' knowledge. They had to decide whether or not the conclusion logically followed from the premises (by pressing associated keys on a keyboard). Most important, while the content of the problems always concerned stock trading, the plausibility of the inference problems was systematically varied. Half of the problems contained a conclusion that was highly plausible for stock-brokers while the other half led to a highly implausible one. Four groups of inferences were obtained by combining logicality and plausibility: “valid-plausible”, “invalid-implausible”, “valid-implausible”, and “invalid-plausible”. Note the conflict between logicality and plausibility in the latter two types, while no such conflict appeared in the first two (Figure 1 bottom). Plausibility of the conclusions was related to the rules of calculating stock prices at the Frankfurt stock exchange (http://www.boerse-frankfurt.de/DE/index.aspx?pageID=44&NewsID=99). Neutral problems (valid/invalid) were included as controls. The main question of interest was ‘what goes on in the mind of a stock-broker when a conclusion is logically correct, but conflicts with what the stock-brokers believes to be a correct deduction (valid-implausible) or is logically incorrect but highly plausible (invalid-plausible)?’

### Experiment 2

#### Participants

As a control, a group of 19 meteorologists from the German Meteorological Service was tested. They were matched (age, education, expertise in their profession) to the group of stock-brokers. They were all naïve with respect to the stock exchange market. All provided informed written consent.

#### Materials and procedure

Inference problems, task, and procedure were identical to Experiment 1.

## Results

### Experiment 1

**Figure 2:**
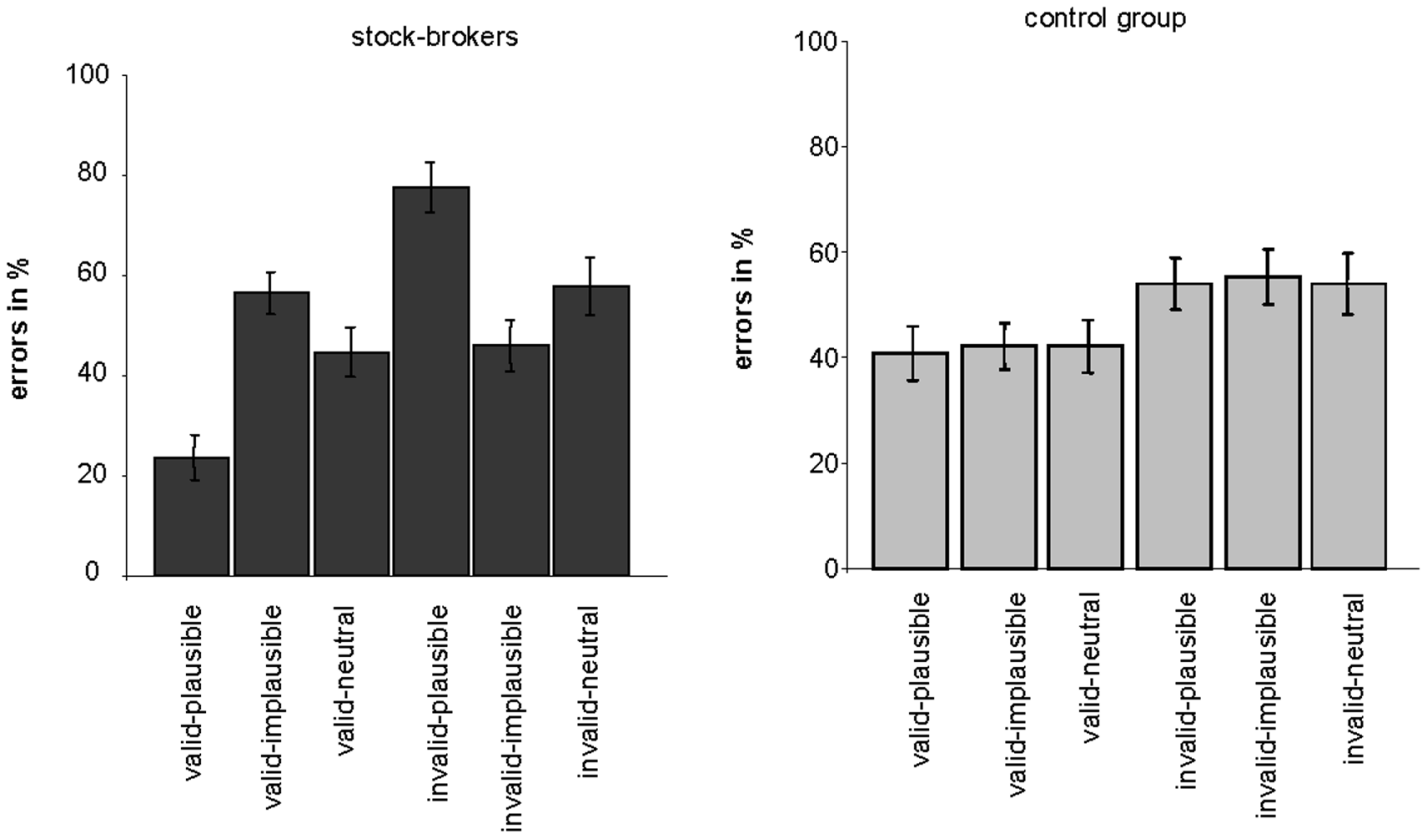
Results of Experiment 1 and 2. Error rates for the stock-brokers (left) and the control group of meteorologists (right). Error bars denote the standard errors (SEM).

Overall, almost half of the problems were solved incorrectly (51% errors overall). A 3×2 ANOVA was run to test our hypotheses that there should be a main effect of validity and–most importantly–an interaction between validity and plausibility. This analysis indeed revealed a significant main effect of Validity, F(1,18) = 15.684, p<.001, indicating that stock-brokers made more errors in evaluating invalid than valid problems, and a significant Validity × Plausibility interaction, F(2,36) = 16.951, p<.001 (there was no main effect of Plausibility, F(2,36) = 0.014, p = .986 n.s.). To further test our hypotheses concerning the effect of plausibility on reasoning we then performed paired-samples t-tests as post-hoc tests. These showed that with valid problems, stock-brokers made significantly more errors when the content was implausible (M = 56.6%; SD = 4.20) than when it was plausible (M = 23.7%; SD = 4.47), t (18) = −5.43; p<.01; r = 0.79. With invalid problems, however, stock-brokers made more errors when the content was plausible (M = 77.6%; SD = 5.02) than when it was implausible (M = 42.1%; SD = 5.15), t (18) = 4.15; p<.01; r = 0.69. This shows that the stock-brokers were biased towards accepting logically invalid inferences as valid if the inference was ‘economically’ plausible (Figure 2 left).

### Experiment 2

A 3×2 ANOVA detected a main effect of Validity, F (1,18) = 6.94, p = .017, reflecting a higher error rate with invalid than with valid problems. No significant effect of Plausibility emerged, F(2,36) = 0.084, p = .92 n.s.. Also, there was no significant Validity × Plausibility interaction, F(2,36) = 0.032; p = .969 n.s.. In other words, no bias towards accepting logically invalid inferences as valid –if the inference was ‘economically’ plausible– occurred (Figure 2 right).

### Overall Analysis of the Plausibility Effects

**Figure 3:**
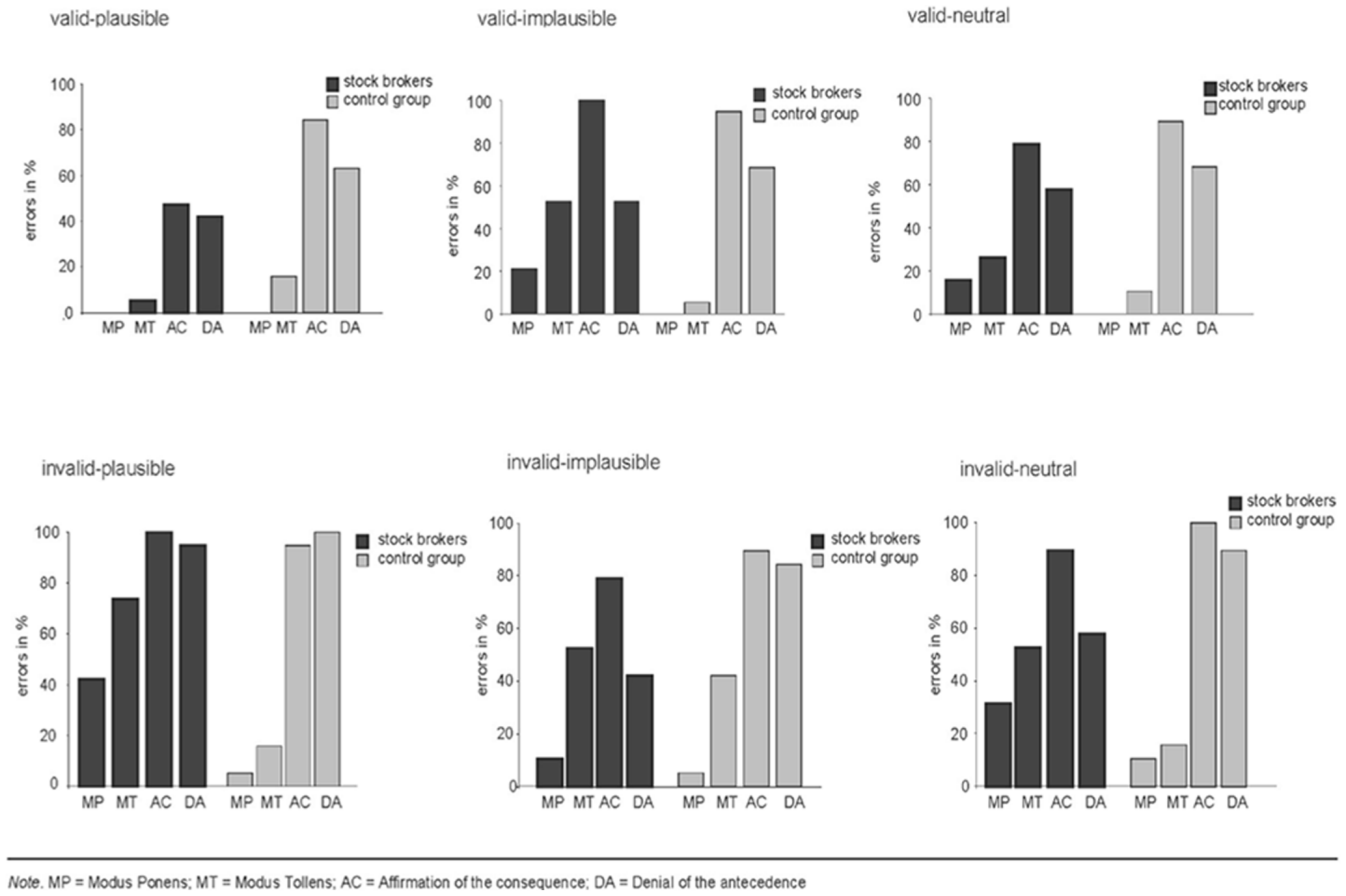
Influence of plausibility. Error rates for the stock-brokers and the control group for the four different inferences (MP, MT, DA, AC) for each condition (valid-plausible, valid-implausible, valid-neutral, invalid-plausible, invalid-implausible, invalid-neutral).

The two experiments only differed in having different groups of participants. The participants of Experiment 1 were experienced stock-brokers, whereas the participants in Experiment 2 served as controls. It is important to see that this actually is not a variation in expertise in the sense that we compared experts to low-experienced stock traders as it is often done in the field of expertise research. In fact, the participants in Experiment 2 primarily served as controls to make sure that the pattern of results in Experiment 1 (stock-brokers) was not caused by other variables in our experimental materials. However, in both experiments, validity and plausibility were used as within-subjects factors and we demonstrated that stock-brokers show a decrement in performance with implausible compared to the plausible inferences, whereas the control group was not affected by the conclusions' plausibility. We did not treat the experiments as a single study with the two different groups of participants as a between-subjects factor, because a conjoint analysis would result in problems with the inhomogeneity of variance. However, a direct interaction between the two groups of participants (brokers and controls) and the different sorts of problems would provide additional support for our account. Therefore, we computed a post hoc ANOVA with “plausibility” as a within-subjects factor and the two experiments as a between-subjects factor. In this way, it is possible to estimate whether the pattern of performance is different for brokers and control participants. Figure 3 summarizes how the plausibility affected reasoning accuracy in the two groups of participants. As indicated by the single experiments, stock-brokers indeed show a significantly different pattern of performance across the different groups of problems. Accordingly, the ANOVA did not show main effects of Validity, F(1,36) = 0.82; p>.05 and Plausibility F(2,72) = 0.00; p>.05, but, there was, as we predicted, a significant interaction between the two factors, F(2,72) = 13.05; p<.01. This shows that the patterns of performance were in fact different for brokers and control participants.

## Discussion

We conducted –in spring 2008, just before the current financial crises started– a study on logical reasoning at the stock market. In particular, we predicted that stock-brokers should show a belief bias whenever there was a conflict between logicality and plausibility. In contrast, we predicted that people with no special experience at the stock market (our control group were meteorologists) should not be sensitive to the implausibility of the reasoning problems' content and thus should not show a belief bias.

Our findings support our hypotheses. Stock-brokers were guided more by prior knowledge and existing beliefs than by logic and rational decision-making. In fact, they often tended to draw logically invalid inferences in favour of their existing beliefs. Thus, they had difficulties to disengage themselves from vastly anchored thinking patterns and instead demonstrated the well-known belief bias [13]. Interestingly, the absence of logical thinking was most clearly noticeable when the stock-brokers were challenged to judge conclusions that were logically incorrect but were in line with their beliefs. In these cases, they made many incorrect decisions, which were also accompanied by longer decision times (not reported here). Their performance was even inferior to that of a control group of meteorologists who had no experience at all with the stock market. However, it should also be mentioned that although the performance of stock-brokers on logical reasoning problems were poor when these tasks caused a conflict between their expert knowledge and the logically valid inference, their performance was actually superior to that of the control group when the inference is both logically valid and in line with their expert knowledge.

How are our results related to different reasoning theories? Our experiments were not designed to test competing reasoning theories and so the following thoughts must be taken with caution. However, there are three possible explanations for why stock-brokers show such a belief bias and thus a strong tendency to deviate from the rules of logic.

(1) The classical heuristics-and-biases program [15] assumes that people use heuristics to deal with the limitations of the cognitive system, leading to systematic errors and lapses of reasoning that point to human irrationality. A modern version of this account is given by the fast and frugal heuristic [16]. Gigerenzer and Goldstein [16] question whether deviations from the formal norms of logic and probability theory must be deemed as “errors“ or “biases”. Within this approach the decisions of our participants may be considered (ecologically) rational to the extent that the heuristics used are adapted to the task environment [17]. Although we agree that this approach can explain some empirical findings, we do not believe that it is helpful in the present context. As Dougherty and others argued the models of fast and frugal heuristics have been too vaguely specified and also do not explain how general-purpose logical skills and domain-specific knowledge (or cues) interact [18].

(2) Our findings are also in line with dual-process theories of reasoning. They explain belief biases in reasoning due to the involvement of different cognitive processes and imply a specific relation between logic and human reasoning. Evans [19] claims that two cognitive systems exist of which one is a “logical” system (slow, requires deliberative control; system 2) and the other is “non-logical” (fast, not consciously accessible; system 1). While the logical system is isolated from knowledge and available to conscious reflection, the other is not isolated from knowledge and can lead us into reasoning errors. Belief biases occur because the system 1 dominates system 2 when the problem is related to individuals' prior knowledge or beliefs. However, there are some shortcomings in this theory that prevent us from accepting it as an explanation for our data. For instance, it postulates loose dichotomies of systems without defining the computations that are performed by them [17], [20]. Other severe problems with dual-process accounts are described, for instance, in [21], [22].

(3) What we believe might be the most promising explanation is the theory of preferred mental models [23]–[26]. The preferred models theory is based on the classical mental model theory by Johnson-Laird and collaborators [27], [28], but makes some additional assumptions concerning the construction, inspection, and validation of mental models that capture the state of affairs described in the premises. In both accounts the models are mental simulations of the problem from which a solution can be developed. However, in the classical model theory, it is assumed that reasoners can use their working memory to carry out recursive processes in order to construct all possible mental models (for an evaluation of this account see [29]). The preferred mental model theory, in contrast, disbelieves that people are able to account for all possible models (solutions) a problem might have. Instead, according to the theory, people typically focus on a single model –the preferred mental model– and ignore alternatives which are also logically valid. The reason is that the preferred model is easier to construct and more effortlessly to maintain and to process in working memory [26]. This effect seems to be even stronger in experts, because experts routinely consider a solution (model) that was successful in former situations, but frequently do not again check its validity in the current situation. Therefore, alternatives to the standard solution –the preferred model– are difficult to consider. This saves cognitive capacities, but makes it difficult to flexibly and rationally respond to new tasks with new solutions. This account must be tested in further experiments.

### Corollaries and Consequences

The main motivation for our study was to explore the interaction between prior beliefs and logical reasoning. Our sample consisted of a group of experienced stock-brokers (and a control group) and the task was to evaluate inferences concerning stock trades. Our main finding was a strong belief bias. Stock-brokers were strongly biased by their prior knowledge. Lowest performance was found for decisions in which the problems caused a conflict between logical validity and prior knowledge. Stock-brokers tended to make logically invalid inferences rather than give up their existing beliefs.

We think that these findings also have some implications for the current financial crisis. Of course, such transformations from the psychological lab into the real world are highly speculative and in fact it is questionable whether individuals' belief biases can cause aggregate effects on a macroeconomic level. On the other hand, we also believe that Cognitive Psychology should not completely abandon to apply experimental findings to the real world and to improve our understanding of it. So, our interpretation of the study is that, amongst others, also psychological mechanisms exist that can help us understand some aspects of financial breakdowns. “Greed” probably is a less important factor than many people think. In fact, research from Cognitive Neuroscience has shown that reasoning with familiar and unfamiliar problems is related to specific patterns of brain activity and the belief bias can be seen as a conflict between brain areas in which either domain-general or knowledge-driven reasoning strategies are implemented [7], [9]–[12]. One lesson from our study is that such neuro-cognitive principles that influence human thinking might also be effective at the stock market. So, to accuse the brokers for their “malfunctions” is probably wrong, because such hard-wired principles are difficult to suppress even if the person is aware of them [13], [19], [28], [30]. Moreover, logical reasoning is not the most important competence at the stock market. Domain-specific knowledge and beliefs are what makes an expert successful under normal conditions and maybe the current financial crisis would be much more disastrous without the long-lasting experience of the brokers. A second lesson, however, is that it is naïve to trust in the self-regulation of the financial market. From a psychological point of view, we need effective control mechanisms, since the reasoning of economic people (and all other human beings) is error-prone and often irrational.
